# Psychometric Assessment of the Urdu Version of the Index of Dental Anxiety and Fear

**DOI:** 10.21315/mjms2020.27.2.12

**Published:** 2020-04-30

**Authors:** Huma Farid, Lubna Pasha, Maryam Majeed

**Affiliations:** Margalla Institute of Health Sciences, Rawalpindi, Pakistan

**Keywords:** dental fear and anxiety, IDAF-4C, Urdu version, psychometric assessment, validity, exploratory factor analysis

## Abstract

**Background:**

The objective of the current study was to adapt the Index of Dental Anxiety and Fear (IDAF-4C) in the Urdu language and measure its validity and reliability.

**Methods:**

Original English questionnaire of IDAF-4C was translated into Urdu language by a panel of dentists and language experts (Urdu and English) followed by critical evaluation, modification and back translation into English language. A final Urdu questionnaire was distributed among 250 patients visiting the Endodontics section at Margalla Institute of Health Sciences (MIHS), Rawalpindi, Pakistan. Cronbach’s alpha was used to determine the reliability of the Index whereas validity was assessed by exploratory factor analysis (EFA). Mean rank scores of IDAF-4C for male and female participants were evaluated using Mann-Whitney U tests (*P* < 0.05).

**Results:**

Of 250 questionnaires, 209 were returned with a response rate of 84%. Cronbach’s alpha for the Urdu version of IDAF-4C was 0.88. Exploratory factor analysis of the IDAF-4C revealed one factor explaining 55.55% of the common variance (Eigenvalue = 4.5). The mean rank scores of all eight items of IDAF-4C were greater for female participants as compared to male participants with a statistically significant association (*P* < 0.05).

**Conclusion:**

The psychometric analysis of the Urdu version of IDAF-4C showed good reliability and consistency compared to the original version as well as other translated versions.

## Introduction

Fear is an emotional response and consists of emotional, cognitive, behavioural and physiological components (1–2). Anxiety and fear are often used interchangeably in literature. Although the emotional and biological response of an individual to both fear and anxiety are similar, but the stimulus in case of anxiety is ill-defined, imaginary or unidentified. The patient becomes anxious due to a learned process from his environment or the imagination of the experience (3). The sight or thought of a dental needle or handpiece can arouse fear or anxiety in an individual and therefore the activation of flight response results in avoidance of dental visits. Thom et al. (4) reported that 80% of adults in the United States are apprehensive about dental treatment and 20% (of these adults) are highly anxious and 5% avoid dental treatment altogether. In a study among adults in Finland, dental fear was found to be associated with 41% of non-habitual dental attendance (5).

Berggren (6) in 1984 presented a vicious cycle of dental fear and anxiety which showed that avoidance of dental visit due to fear and anxiety results in deterioration of oral health status (caries and periodontitis). The deterioration in oral health status is self-reported by the patients as well as demonstrated by clinical indices (7). This deterioration in dental health status and continued fear and anxiety can also initiate feelings of inferiority, shame and embarrassment. Subsequently, these feelings can give rise to social problems like avoidance of social gatherings and contact with people. If dental issues are not managed at this stage, these patterns of effects can lead to higher and more widespread anxiety of dentistry and even phobia. To measure dental fear and anxiety different scales have been used namely Corah’s Dental Anxiety Scale (DAS), Kleinknecht’s Dental Fear Survey (DFS), Stouthard’s Dental Anxiety Inventory, Weiner’s Fear Questionnaire, Fear of Dental Treatment Cognitive Inventory and Modified Dental Anxiety Scale (MDAS) (8, 9). Most of the scales measure fear stimuli rather than fear itself. Even those measuring fear do not account for cognitive, behavioural and physiological components of fear (8), they are also criticised for their poor construction, validity and response rate (2, 9–10). Armfield in 2010 introduced the Index of Dental Anxiety and Fear (IDAF-4C+) for overcoming the shortcomings in already existing indices (11). IDAF-4C measures four components of dental fear, namely emotional, behavioural, physiological and cognitive with two questions related to each component of fear. Dental phobia (IDAF-P) and potential anxiety-inducing stimuli (IDAF-S) are demonstrated by ‘+’. From 2010 till date, this index has been translated in Spanish, German, Swedish, Finnish, Turkish and Malay languages with proven reliability and validity (12–17). Urdu is the national language of Pakistan and patients can understand this language more easily as compared to the English language, therefore we translated IDAF-4C in Urdu. The objective of the current study was to develop the Urdu version of IDAF-4C and measure its validity and reliability among patients visiting Margalla Dental Hospital.

## Methods

### Cross Cultural Adaptation and Ethical Approval

Permission was obtained for Urdu translation of the original English language version of IDAF-4C, from Jason Armfield via email. Ethical approval was taken by the Ethical Review committee of the Margalla Institute of Health Sciences (MIHS), Rawalpindi, Pakistan. Written consent was taken from the participants included in this study explaining the purpose of the study as well as their right to participate or not to participate in the study.

Translation into the Urdu language was carried out according to guidelines proposed by Siny Tsang and colleagues (18). The original English version of IDAF-4C questionnaire was translated into the Urdu language by a person expert in both English and Urdu languages and had experience in translations. This Urdu questionnaire was then back translated into the English language by another person who was blinded to the original English questionnaire and objective of the study. These questionnaires were then reviewed by a panel of dentists (authors and senior dentists) and language experts. After making corrections, a second version of the Urdu translation was generated. There were disagreements between language experts and subject specialists on few words in Urdu version. To resolve the discrepancies and develop a consensus, modifications were made in the Urdu version followed by back translation for the second time into English language. Both Urdu and English versions were viewed and agreed upon by all the dentists to be distributed among study subjects.

The questionnaire had two sections. The first section covered questions related to demographic details of the participants (age, gender, education, monthly income). The second section covered eight items IDAF-4C core module with two questions related to each component of fear and anxiety. Each item had five possible responses ranging from Disagree (score 1) to Strongly agree (score 5). Those with an IDAF-4C mean score less than 1.49 were considered as having No to Low fear, 1.50–2.49 as Low to Moderate fear, 2.50–3.49 as Moderate to High fear and > 3.50 were considered as having Extreme fear (11, 12, 14).

### Sample Size Estimation

Exploratory factor analysis (EFA) was used to determine the validity of the Urdu version of IDAF-4C. Stevenson suggested the minimum sample size of five subjects per variable for exploratory factor analysis (19). As IDAF-4C has eight items, the minimum sample size turned out to be 40.

Cronbach’s alpha was used to determine reliability. With power (1-β) of study set at 0.8, level of significance (α) at 0.05 and lowest expected Cronbach’s alpha at 0.7, (StatsToDo.com) sample size turned out to be 201 (20).

Taking into consideration sample size required for validity and reliability, a total of 250 questionnaires in Urdu language were distributed among patients visiting the Endodontics section of the Operative Dentistry Department at MIHS.

### Inclusion and Exclusion Criteria

Patients visiting for root canal treatment between the age group of 18–60 years and were able to read and write the Urdu language were included in the study. Patients suffering from systemic diseases and taking medications were excluded from the study.

### Statistical Analysis

SPSS 20 was used for data analysis. Cronbach’s alpha was used to determine the reliability by internal consistency. EFA with principal axis factoring was used to recognise more than one underlying construct within the measure. Factors extraction was based on Eigenvalues more than 1. Factor loading was kept more than 0.4. Mean and median scores of all eight items of IDAF-4C were assessed. Mean rank scores of IDAF-4C between male and female participants were evaluated with Mann-Whitney U tests (*P* < 0.05).

## Results

Out of 250 questionnaires, 209 were returned with a response rate of 84%. The mean age of participants was 31.8 years with 103 (49%) female and 106 (51%) male. Demographic variables in detail are shown in Table 1.

According to the IDAF-4C, of the participants 52.2% had No to Low fear (score 1–1.49), 24.4% had Low to Moderate fear (score 1.5–2.49), 18.7% had Moderate to High fear (score 2.5–3.49) and 1.2% had High to Extreme dental fear (score > 3.50) (Table 2).

Cronbach’s alpha for the Urdu version of IDAF-4C was 0.88. Corrected item-total correlations of the IDAF-4C ranged from 0.54 to 0.73. Table 3 shows the analysis and reliability of each item of IDAF-4C.

**Figure 1:**
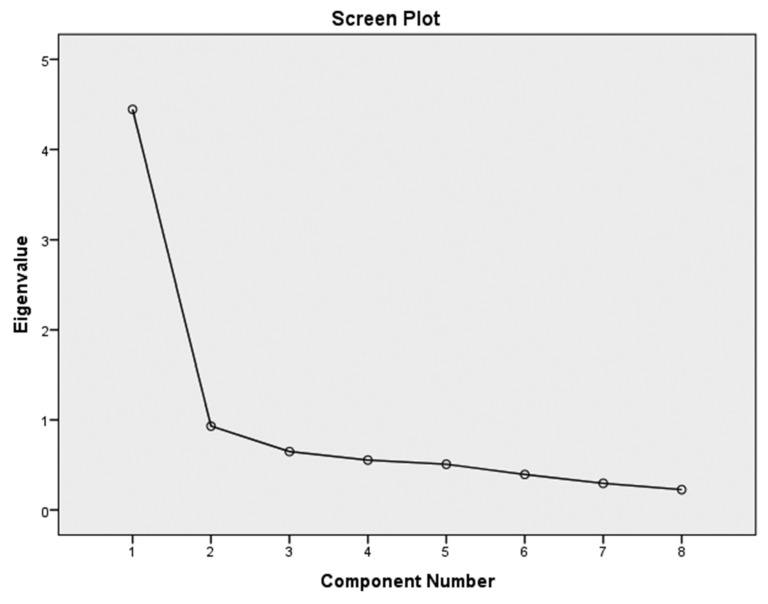
Screen plot for EFA Note: EFA revealed single factor (Eigenvalue = 4.5) explaining 55.55% of common variance

Kaiser-Meyer-Olkin (KMO) measure of sampling adequacy was 0.871, approving the adequacy of data for factor analysis. Bartlett’s test of sphericity was significant (*P* < 0.01). EFA of the IDAF-4C revealed one factor explaining 55.55% of the common variance (Eigenvalue = 4.5). This means that all items of the scale are closely related to each other and measure the same. Figure 1 shows the attribution of scale has been gathered in one factor higher than 1.

Median (interquartile range [IQR]) and mean (standard deviation [SD]) values for all eight items IDAF-4C are listed in Table 4. Mean rank scores according to gender are also listed in Table 4. The mean rank scores of all eight items of IDAF-4C were greater for females as compared to males. This difference was statistically significant (*P* < 0.05).

## Discussion

According to the results of our study majority (52%) of individuals had No to Low fear of dental treatment. Finnish version (14) of IDAF-4C showed 61.6% individuals with No fear of dental treatment, whereas the Australian population (8) showed that 51% individuals had No to Low fear. The differences in results can be attributed to different study subjects. In our study, the sample was taken from individuals already visiting the hospital for dental treatment, whereas in Finnish study, the subjects were dental students. Although our result is similar to the Australian study yet their sample was taken from the general population.

Cronbach’s alpha value in our study was 0.88 suggesting high reliability of the scale as well as high internal consistency of scale items. The reliability coefficient greater than 0.80 is considered ‘good’ and greater than 0.90 is considered ‘excellent’ in most social science research citations (21–23). Our results are comparable with the Finnish version (14) and the original version (11) having Cronbach’s alpha of 0.88 and 0.91, respectively. Higher reliability was also found in Spanish and Turkish version (12, 16) with Cronbach’s alpha of 0.94 and 0.96, respectively.

EFA revealed a single factor (Eigenvalue = 4.5) explaining 55.55% of common variance. Finnish and Turkish versions, yielded one factor with 51.7% (Eigenvalue 4.6) and 79.72% (Eigenvalue = 6.38) of common variance respectively (14, 16). These results showed that all eight items in the index were one-dimensional and homogenous, and each item measured the same as other items in the index. Spanish and Swedish versions also showed clear one-dimensional nature of IDAF-4C (12, 15).

Mean rank scores of all eight items of IDAF-4C were higher for females as compared to males with statistically significant association. This was in line with previous studies on dental fear (using IDAF-4C or other fear indexes) showing that the females were more anxious to dental treatment as compared to males (14, 16, 24–26). Physiological conditions such as fear, stress, pain and social phobia are more common in females and they exhibit high levels of neuroticism (26). Also, males are usually less expressive in their feelings and tend to express fear not as readily as females do (25–26). Still, some studies are showing no significant differences between gender and dental fear and anxiety (27–28).

Standardised and validated research instrument (scale/questionnaire) enables the comparison of results of different studies across the globe. It also increases the certainty with which the instruments accurately reflect what they are supposed to measure (29–30). To use an instrument in another language, setting and time, cross-cultural adaptation is important. Cross-cultural adaptation reduces the risk of bias in the study as well as ensure retention of validity and reliability of each item in the scale (30). Cross-cultural adaptation was carried out in our study by translation of the questionnaire into the Urdu language by language experts followed by a back-translation into the English language by a third person not aware of the primary questionnaire. At each step, all the items on the questionnaire were critically evaluated by the expert team and changes were made accordingly so that the translation process retains the actual spirit of the study as well as adapts to Pakistani culture.

## Conclusion

The psychometric analysis of the Urdu version of IDAF-4C showed good reliability and consistency compared to the original version as well as other translated versions. Nearly half of the population showed No to Low fear with females experiencing more fear and anxiety as compared to males. The results of this study cannot be generalised to the entire Pakistani population and there is a need to measure fear and anxiety using the IDAF-4C scale on the community level. Studies should also be formulated comparing results of the Urdu version of IDAF-4C with other established fear and anxiety scales.

## Figures and Tables

**Table 1:** Demographic details of the study participants

Variables	Number (%)
Gender	
Male	106 (51)
Female	103 (49)

Total	**209 (100)**

Monthly income (Pakistani Rupees)	
16,000–25,000	66 (31.7)
26,000–35,000	52 (24.6)
36,000–50,000	41 (19.7)
Greater than 50,000	50 (24.0)

Total	**209 (100)**

Educational status	
Less than 10 years	47 (22.5)
Matriculation	22 (10.5)
Intermediate	40 (19.1)
Graduation	75 (35.9)
Post-graduation	25 (12.0)

Total	**209 (100)**

**Table 2:** IDAF- 4C score of the participants and distribution according to gender

Score of participant	Total = 209 *n* (%)	Male (106) *n* (%)	Female (103) *n* (%)
No to Low fear (1.0–1.49)	109 (52.2)	68 (62.3)	41 (37.6)
Low to Moderate fear (1.50–2.49)	51 (24.4)	20 (39.2)	31 (60.7)
Moderate to High fear (2.50–3.49 )	39 (18.7)	13 (33.3)	26 (66.6)
Extreme fear (> 3.50)	10 (4.7)	5 (50)	5 (50)

**Table 3:** Item analysis and reliability of the IDAF-4C (*n* = 209)

IDAF-4C items	Corrected item-total correlation	Cronbach’s alpha if item deleted
I feel anxious shortly before going to the dentist	0.59	0.87
I generally avoid going to the dentist because I find the experience unpleasant or distressing	0.67	0.86
I get nervous or edgy about upcoming dental visits	0.73	0.85
I think that something really bad would happen to me if I were to visit a dentist	0.65	0.86
I feel afraid or fearful when visiting the dentist	0.73	0.86
My heart beats faster when I go to the dentist	0.72	0.86
I delay making appointments to go to the dentist	0.54	0.87
I often think about all the things that might go wrong prior to going to the dentist	0.55	0.87

**Table 4:** Median (IQR), mean (SD) and mean rank scores of eight items of IDAF-4C according to gender

IDAF-4C	Median (IQR)	Mean (SD)	Male	Female	*P*-value*

			Mean rank (*n* = 106)	Mean rank (*n* = 103)	
I feel anxious shortly before going to the dentist	1.0 (2.0)	1.94 (1.21)	86.88	123.65	0.000
I generally avoid going to the dentist because I find the experience unpleasant or distressing	1.0 (2.0)	1.75 (1.04)	97.63	112.59	0.042
I get nervous or edgy about upcoming dental visits	1.0 (1.0)	1.75 (1.07)	97.12	113.11	0.031
I think that something really bad would happen to me if I were to visit a dentist	1.0 (1.0)	1.61 (1.07)	97.88	112.33	0.036
I feel afraid or fearful when visiting the dentist	1.0 (1.0)	1.69 (1.05)	87.53	122.98	0.000
My heart beats faster when I go to the dentist	1.0 (1.0)	1.71 (1.21)	96.78	113.46	0.018
I delay making appointments to go to the dentist	1.0 (2.0)	1.92 (1.19)	96.29	113.97	0.020
I often think about all the things that might go wrong prior to going to the dentist	1.0 (1.0)	1.58 (0.97)	94.99	115.31	0.004

Notes:

* Mann-Whitney U test (level of significance *P* ≤ 0.005);

IQR = interquartile range; SD = standard deviation
